# A multicenter cohort study on the efficacy, retention, and tolerability of cenobamate in patients with developmental and epileptic encephalopathies

**DOI:** 10.1111/epi.18308

**Published:** 2025-02-11

**Authors:** Elisa Buhleier, Susanne Schubert‐Bast, Susanne Knake, Felix von Podewils, Hajo M. Hamer, Nico Melzer, Gerhard Kurlemann, Kerstin Alexandra Klotz, Laurent M. Willems, Felix Rosenow, Andreas Brunklaus, Adam Strzelczyk

**Affiliations:** ^1^ Epilepsy Center Frankfurt Rhine‐Main, Department of Neurology University Medicine Frankfurt, Goethe University Frankfurt Frankfurt am Main Germany; ^2^ Department of Pediatrics, Pediatric Epileptology Division University Medicine Frankfurt, Goethe University Frankfurt Frankfurt am Main Germany; ^3^ Epilepsy Center Hessen, Department of Neurology Philipps University Marburg Marburg Germany; ^4^ Department of Neurology, Epilepsy Center University Hospital Greifswald Greifswald Germany; ^5^ Epilepsy Center, Department of Neurology, University Hospital Erlangen Friedrich Alexander University Erlangen‐Nuremberg Erlangen Germany; ^6^ Department of Neurology, Medical Faculty and University Hospital Heinrich Heine University Düsseldorf Düsseldorf Germany; ^7^ St. Bonifatius Hospital Lingen Germany; ^8^ Department of Pediatric Neurology University Hospital Bonn Bonn Germany; ^9^ Department of Neuropediatrics and Muscle Disorders, Center for Pediatrics, Medical Center, Faculty of Medicine University of Freiburg Freiburg im Breisgau Germany; ^10^ School of Health and Wellbeing University of Glasgow Glasgow UK; ^11^ Paediatric Neurosciences Research Group Royal Hospital for Children Glasgow UK

**Keywords:** antiseizure medication, Dravet syndrome, epilepsy, Lennox–Gastaut syndrome, seizure

## Abstract

**Objective:**

This study was undertaken to evaluate retention and treatment characteristics of cenobamate (CNB) in patients with developmental and epileptic encephalopathies (DEEs) in clinical practice.

**Methods:**

This multicenter, retrospective cohort study recruited all patients with DEEs who started CNB treatment between October 2020 and April 2023 at participating epilepsy centers.

**Results:**

A total of 41 patients (mean age = 28.3 ± 13.1 years, median = 26 years, range = 4–73 years; 24 male [58.5%]) were treated with CNB. Of these, 33 had Lennox–Gastaut syndrome, seven had tuberous sclerosis complex, and one had Dravet syndrome. The median number of antiseizure medications (ASMs) at enrollment was three, and patients had a median of eight failed ASMs in the past. The retention rate for CNB was 94.9% at 3 months, 82.9% at 6 months, and 72.4% at 12 months of follow‐up. Cumulative exposure to CNB was 477 months (39.2 years). Efficacy (50% responder rate) at 3 months was 39% including 7.3% seizure‐free patients. Long‐term, the 50% responder rate at 12 months was 34.5% (seizure‐free [10.3%]). There was no difference in response at 3 months regarding sex, age (adult vs. children), previous and concomitant number of ASMs, or first target dose of CNB. Treatment‐emergent adverse events were predominantly sedation and dizziness and were observed in 58.5% of patients. Children and adolescents showed comparable efficacy, retention, and tolerability compared to adults.

**Significance:**

The findings from this open‐label, retrospective study suggest that CNB may be effective in some patients with DEEs. Its overall use in DEEs seems to be safe and well tolerated. We observed similar response, retention, and adverse event profiles in children and adults.

**Key points**

- Treatment with cenobamate was studied in 41 patients with developmental and epileptic encephalopathies.
- Retention rate was 94.9% at 3 months, 82.9% at 6 months, and 72.4% at 12 months of follow‐up.
- Fifty percent responder rate was 39% (7.3% seizure‐free) at 3 months and 34.5% (10.3% seizure‐free) at 1 year.
- Eight children and adolescents (mean age = 13 years) showed comparable efficacy, retention, and tolerability compared to adults.
- Treatment‐emergent adverse events were predominantly sedation and dizziness and were observed in 58.5% of patients.

## 1 INTRODUCTION

Cenobamate (CNB) is a novel antiseizure medication (ASM) that was approved in 2019 in the United States and 2021 in the EU as adjunctive therapy for adults with focal onset seizures with or without secondary generalization.^1^, ^2^, ^3^ Several clinical trials have shown promising results regarding efficacy, tolerability, and safety.^3^, ^4^, ^5^, ^6^ Although its mechanism of action is not fully understood, it is believed to involve a dual pathway, with CNB blocking voltage‐gated sodium channels and enhancing γ‐aminobutyric acid‐mediated inhibition, leading to a reduction in neuronal excitability.^7^, ^8^

Developmental and epileptic encephalopathies (DEEs) are a heterogeneous group of epilepsy syndromes, in which the epileptic activity itself contributes to progressive cognitive and behavioral impairment beyond what might be expected from the underlying structural or genetic pathology alone.^9^ The onset usually occurs in infancy or early childhood and encompasses several well‐defined epilepsy syndromes, such as Dravet syndrome (DS) or Lennox–Gastaut syndrome (LGS).^10^, ^11^ These epilepsy syndromes are characterized by their age at onset, seizure types, electroencephalographic patterns, developmental trajectory, and underlying etiology.^9^ Most affected patients suffer from a refractory course of disease, with frequent seizures and diverse seizure types. Frequent hospitalizations, as well as an increased need for medical and nursing care, place a significant social, interpersonal, and economic burden on patients, caregivers, and society.^12^, ^13^

The objective of this multicenter study was to evaluate the efficacy, retention, and tolerability of CNB in patients with DEEs.

## 2 MATERIALS AND METHODS

This study was performed at seven German epilepsy centers (Düsseldorf, Erlangen, Frankfurt am Main, Freiburg im Breisgau, Greifswald, Marburg, and Münster/Lingen). All patients with DEEs treated with CNB at one of the enrolling epilepsy centers between October 2020 and April 2023 were included. DEE diagnosis was based on the International League Against Epilepsy classification^10^, ^11^ and the consensus statement of the International TSC Consensus Group.^14^ The retrospective analysis was approved by the ethics committee of the University of Frankfurt. Informed consent was waived due to the retrospective nature of the study design, and STROBE (Strengthening the Reporting of Observational Studies in Epidemiology) guidelines were followed.^15^ The study was not sponsored or funded by any third party.

Patients' data were collected through their medical records and included etiology, sex, age at epilepsy onset and start of CNB treatment, seizure frequency in the 3 months prior to CNB treatment (defined as baseline), prior and concomitant treatment with other ASMs, dose and titration of CNB (initial dose, target dose, and maximum dose), and intellectual disability (none/not reported, mild, moderate, or severe). The term *target dose* refers to the dose initially aimed for during the titration process, as determined by the treating physician to minimize the risk of adverse events. Follow‐up data, also collected through patients' medical records, included target and maximal doses of CNB, seizure frequency, physician‐rated Clinical Global Impression of Change (CGI‐C), treatment‐emergent adverse events (TEAEs), retention of CNB, and discontinuation of CNB for the following reasons: TEAEs, a lack of effectiveness, both TEAEs and a lack of effectiveness, or not reported.

Seizure reduction was analyzed at 3‐, 6‐, and 12‐month follow‐ups for total seizures and generalized tonic–clonic seizures (GTCS), where data were available. Total seizures combined generalized tonic–clonic, tonic, atonic, atypical absence, myoclonic, and focal impaired awareness seizures. A 25% responder rate was defined as 25% or greater seizure reduction compared to the defined baseline. A 50% responder rate meant a 50% or greater seizure reduction, and a 75% responder rate a 75% or greater seizure reduction; 100% response was classified as seizure‐free compared to baseline. No response was defined as a change (decrease or increase) in seizure frequency by less than 25% compared to baseline. Seizure increase was defined as a 25% or greater increase in seizure frequency compared to baseline. In addition, change in seizure occurrence was recorded as an average of seizure days per month regardless of seizure type at baseline and last follow‐up. Physicians rated overall clinical change (CGI‐C) during treatment with CNB on a 7‐point rating scale, categorized from very much improved to very much worse. Retention rate was defined as patients continuing CNB treatment after 3, 6, and 12 months and was estimated using Kaplan–Meier survival curves. TEAEs were classified using the categories central nervous system (CNS)/ataxia, behavior, skin, and other. Data acquisition was performed using standardized and anonymized reporting forms.

The statistical analyses described above, including the descriptive analyses, were performed using IBM SPSS Statistics, version 28. Retention time was displayed using Kaplan–Meier survival curves. Chi‐squared and log‐rank tests were used for statistical analysis, and *p*‐values < .05 were regarded as statistically significant.

## 3 RESULTS

### 3.1 Patient demographics at baseline

A total of 41 patients (58.5% male) with a mean age of 28.3 years (SD = ±13.1, median = 26 years, range = 4–73), including eight children and adolescents younger than 18 years (19.5%, mean age = 13 years), were enrolled in the study.

The underlying epilepsy syndromes or etiologies included LGS (*n* = 33, 80.5%), tuberous sclerosis complex (TSC; *n* = 7, 17.1%), and DS (*n* = 1, 2.4%). Among those diagnosed with LGS, 14 patients had an underlying structural etiology, and six patients presented with pathogenic variants in genes including *COL4A2*, *GPHN*, *HUWE1*, and *SZT2*, a 2q37 deletion, and a chromosomal aberration in chromosome 9 (each *n* = 1).

The mean epilepsy duration at study entry was 22.2 ± 12.6 years (median = 19), with an epilepsy onset at a mean age of 6.0 ± 9.6 years (median = 2). The mean ASM number at enrollment was 3.46 ± 1.0 (median = 3.0, range = 2–6 ASMs). Lamotrigine (*n* = 20, 48.8%, mean dose = 341 mg), brivaracetam (*n* = 15, 36.6%, mean dose = 268 mg), valproate (*n* = 14, 34.1%, mean dose = 1379 mg), clobazam (*n* = 14, 34.1%, mean dose = 10.5 mg), lacosamide (*n* = 11, 26.8%, mean dose = 386 mg), perampanel (*n* = 11, 26.8%, mean dose = 6.9 mg), cannabidiol (*n* = 10, 24.4%, mean dose = 805 mg), zonisamide (*n* = 5, 12.2%, mean dose = 305 mg), oxcarbazepine (*n* = 4, 9.8%, mean dose = 1875 mg), topiramate (*n* = 4, 9.8%, mean dose = 325 mg), and rufinamide (*n* = 4, 9.8%, mean dose = 2500 mg) were the most frequently prescribed drugs prior to the start of CNB treatment (Figure S1A); everolimus was used for the treatment of one patient with TSC. The patients reported a mean number of 7.9 ± 3.6 ASMs (median = 8, range = 2–16) that had failed previously (not including current ASMs). The five most reported prior drugs were levetiracetam (*n* = 31, 75.6%), lamotrigine (*n* = 21, 51.2%), oxcarbazepine (*n* = 21, 51.2%), valproate (*n* = 20, 48.8), and topiramate (*n* = 20, 48.8%); for details, please refer to Figure S1B.

### 3.2 Dosing with CNB

Initial CNB dose varied between 12.5 and 25 mg (mean = 13.1 ± 2.7 mg, median = 12.5 mg). The mean target dose was achieved within a median time of 70 days (mean = 60.4 ± 21.1 days) and ranged between 50 and 300 mg (mean = 150.6 ± 59 mg, median = 175 mg). For the majority of patients, 100 mg (*n* = 16, 39%) or 200 mg (*n* = 19, 46.3%) was chosen as the target dose. Among children and adolescents, the mean target dose ranged between 75 and 300 mg (mean = 121.9 ± 72.5 mg, median = 100 mg), 100 mg in six of eight children (75%) was predominantly selected for younger patients, whereas 200 mg was the target dose in 19 adults. The maximum daily dose of CNB varied between 50 and 400 mg (mean = 206.1 ± 117.5 mg, median = 200 mg), with seven patients taking the upper approved dosage of 400 mg.

### 3.3 Efficacy: Seizure‐free patients and responder rates

During the first 3 months of follow‐up, responder rates for the total seizure count were available for 41 patients. Three patients (7.3%) experienced seizure freedom, 10 (24.4%) had a 75% response rate, 16 (39.0%) had a 50% response rate, and 23 (56.1%) had a 25% response rate. No change was reported by 16 (39.0%) patients, and one reported seizure increase. One patient discontinued the medication. Table 1 shows details for responders and nonresponders regarding sex, age, epilepsy syndrome and etiology, previous and concomitant number of ASMs, and target dose of CNB. No significant differences were found in the chi‐squared analysis.

**TABLE 1:** Clinical characteristics and outcome on follow‐up at 3 months.

Characteristic	All patients, *n* = 41, *n*	Nonresponders, *n* = 18, *n* (%)	Responders, *n* = 23, *n* (%)
Total	41	18 (43.9)	23 (56.1)
Sex			
Male	24	9 (37.5)	15 (62.5)
Female	17	9 (52.9)	8 (47.0)
Age range			
<18 years	8	3 (37.5)	5 (62.5)
≥18 years	33	15 (45.5)	18 (54.5)
Epilepsy syndrome and etiology			
Lennox–Gastaut syndrome	33	14 (42.4)	19 (57.6)
Tuberous sclerosis complex	7	4 (57.1)	3 (42.9)
Dravet syndrome	1	0	1 (100)
Target CNB dosage			
Low dose of ≤150 mg	21	8 (38.1)	13 (61.9)
High dose of ≥200 mg	20	10 (50.0)	10 (50.0)
Previously failed ASMs [without current]			
≤5 ASMs	14	9 (64.3)	5 (35.7)
≥6 ASMs	27	9 (33.3)	18 (66.7)
Number of concomitant ASMs at start of CNB			
2	7	3 (42.9)	4 (57.1)
≥3	34	15 (44.1)	19 (55.9)

Abbreviations: ASM, antiseizure medication; CNB, cenobamate.

**FIGURE 1:**
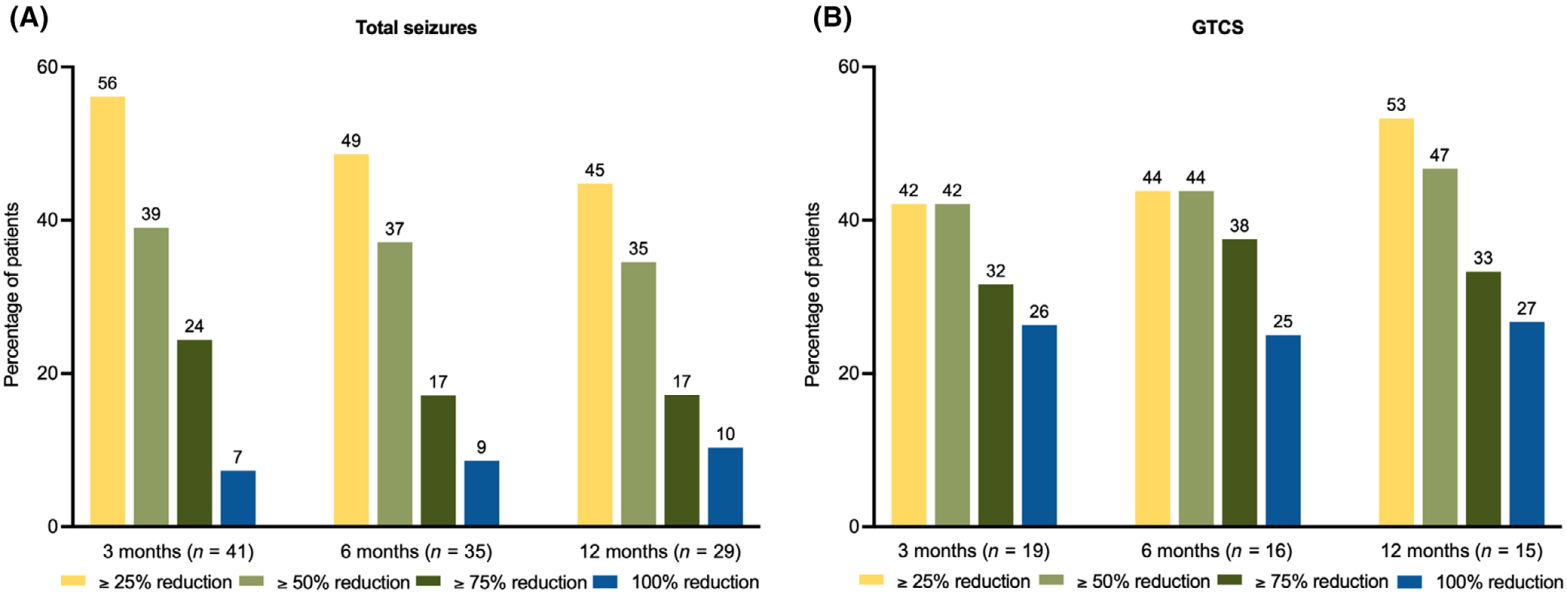
Responder rates over time for (A) total seizures and (B) generalized tonic–clonic seizures (GTCS).

At 6‐month follow‐up, responder rates were available for 35 patients; three (8.6%) patients were seizure‐free, six (17.1%) reported a 75% response rate, 13 (37.1%) had a 50% response rate, and 17 (48.6%) reported a 25% response rate. No change was reported by 11 (31.4%), and one reported seizure increase. Six patients discontinued the medication. At 12‐month follow‐up, responder rates were available for 29 patients; three (10.3%) patients were seizure‐free, a 75% response rate was reported by five patients (17.2%), a 50% response rate was reported by 10 patients (34.5%), and a 25% response rate was reported by 13 patients (44.8%). No change was reported by seven (24.1%), and one reported seizure increase. Eight patients discontinued CNB. For details, please refer to Figure 1A.

Data on GTCS frequency responder rates were available for 19 patients at 3 months; 22 patients had not experienced GTCS in the 12 months before starting CNB. Of the 19 patients, five patients (26.3%) reported seizure freedom from GTCS, six (31.6%) reported a 75% GTCS response rate, and eight (42.1%) reported a 50% GTCS response rate. No change was reported by eight patients (42.1%), and two reported an increase in GTCS. One patient discontinued CNB. After 6 months of follow‐up, GTCS responder rates were available for 16 patients. Four (25.0%) reported no GTCS, six (37.5%) reported a 75% response rate, and seven (43.8%) reported a 50% response rate. No change was reported by five (31.2%), and one reported a GTCS increase. Three patients discontinued CNB. After 12 months of CNB treatment, GTCS responder rates were available for 15 patients; four patients reported seizure freedom from GTCS (26.7%), a 75% response rate was reported by five patients (33.3%), a 50% response rate was reported by seven patients (46.7%), and a 25% response rate was reported by eight patients (53.3%). No change was reported by two (13.3%), and five patients discontinued CNB. For details, please refer to Figure 1B.

Detailed analysis between children and adults did not reveal any differences in responder rates for total seizures or GTCS response.

The patient with DS was an adult (35 years old at the start of CNB treatment, pathogenic *SCN1A* variant) and was a 50% responder for both total seizure count and GTCS at 3, 6, and 12 months. She continued CNB beyond 400 days of treatment in combination with valproate, lamotrigine, perampanel, and ethosuximide.

### 3.4 Seizure days

**FIGURE 2:**
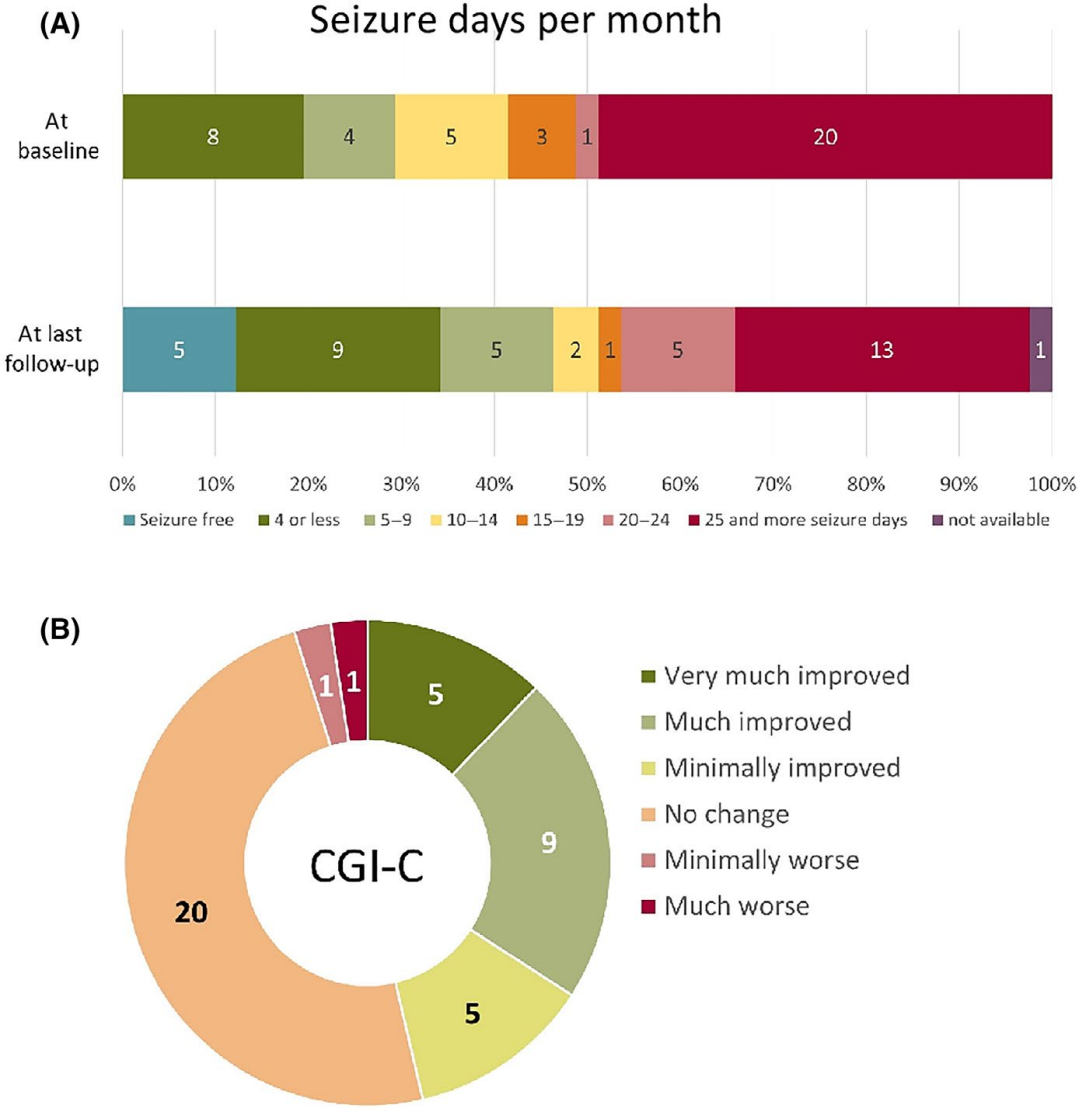
(A) Percentage of patients according to seizure days per month across seven incremental categories at baseline and at the final follow‐up after initiation of cenobamate. (B) Physician‐assessed Clinical Global Impression of Change (CGI‐C).

At baseline, the patients had a mean of 18.5 ± 11.9 seizure days per month (median = 20, range = .17–30) during the 3‐month baseline phase. At the final follow‐up point, the mean significantly decreased to 14.5 (median = 13, range = 0–30) seizure days per month in the last 3 months of CNB treatment (*p* = .008). Figure 2A shows the number of seizure days per month at baseline and final follow‐up. There was no difference in seizure day reduction between children and adults.

### 3.5 Retention and overall change

**FIGURE 3:**
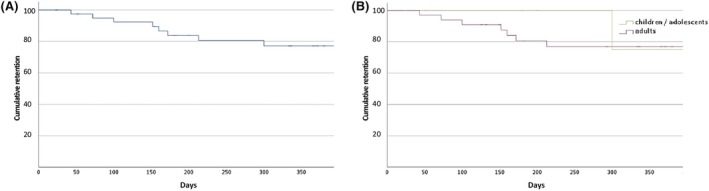
Retention rate of cenobamate (A) in the complete cohort and (B) stratified for adults and children or adolescents (log‐rank *p*‐value = .623).

In total, 10 patients (24.3%) discontinued CNB during the study period. Kaplan–Meier survival curves show the retention over time in Figure 3. The probability of remaining on CNB treatment for all patients was 94.9% at 3 months, 82.9% at 6 months, and 72.4% at 12 months. Cumulative exposure to CNB was 477 months (39.2 years). The reasons for discontinuation of CNB were TEAEs (*n* = 5, 12.2%), insufficient efficacy (*n* = 3, 7.3%), or both (*n* = 2, 4.9%). TEAEs associated with discontinuation were sedation (*n* = 3), behavioral problems (*n* = 2), dizziness (*n* = 1), and rash (*n* = 1). There was no difference in retention between adults and children or adolescents (log‐rank *p* = .623).

Using the CGI‐C, five (12.2%) patients were rated as very much improved at the last follow‐up, nine (22%) patients were much improved, and five (12.2%) patients were minimally improved. Twenty (48.8%) patients showed no change. One (2.4%) patient was rated minimally worse, and one (2.4%) was rated much worse. No patients were rated as very much worse (Figure 2B).

### 3.6 Adverse events

During CNB treatment, 24 (58.5%) patients experienced TEAEs. Most common TEAEs were CNS symptoms (*n* = 19, 46.3%) such as sedation (*n* = 13, 31.7%), dizziness (*n* = 5, 12.2%), ataxia (*n* = 4, 9.8%), cognitive deficits (*n* = 4, 9.8%), visual impairment (*n* = 4, 9.8%), and dysarthria (*n* = 1, 2.4%). Five (12.2%) patients showed behavioral TEAEs, including aggression (*n* = 3, 7.3%), depressive mood (*n* = 2, 4.9%), and agitation (*n* = 1, 2.4%). One patient (2.4%) reported a skin rash/pruritus (CNB discontinued at day 43), and a further two (4.9%) patients showed other adverse events. For details, please refer to Table 2.

**TABLE 2:** Characteristics of TEAEs and their frequency.

TEAE	*n* (%)
Overall occurrence of TEAEs	24 (58.5)
CNS symptoms	19 (46.3)
Sedation	13 (31.7)
Dizziness	5 (12.2)
Ataxia	4 (9.8)
Cognitive deficits	4 (9.8)
Visual impairment	4 (9.8)
Dysarthria	1 (2.4)
Psychobehavioral	5 (12.2)
Aggression	3 (7.3)
Depressive mood	2 (4.9)
Agitation	1 (2.4)
Skin	1 (2.4)
Pruritus/rash	1 (2.4)
Other	2 (4.9)

Abbreviation: TEAE, treatment‐emergent adverse event.

## 4 DISCUSSION

This study presents a multicenter German dataset on the efficacy and tolerability of CNB in a cohort of 41 children, adolescents, and adults with DEEs, a severely affected subgroup typically characterized by drug‐resistant epilepsy and high seizure frequency.^16^, ^17^ These patients face a significant burden, impacting not only their quality of life but also that of their caregivers and society as a whole. DEEs remain an important area of epilepsy research, and novel ASMs, such as cannabidiol and fenfluramine,^18^, ^19^, ^20^ have been developed for specific subgroups of DEE patients including patients with DS, LGS, and TSC. However, there remains limited information on the efficacy and tolerability of CNB in this population.

With a cumulative CNB exposure of 477 months and follow‐up periods of up to 12 months in the majority of patients, this cohort provides valuable insights into the use of CNB for DEEs. Retention rates at 3 (95%), 6 (83%), and 12 months (72%) align with findings from postmarketing studies on CNB and other ASMs—including levetiracetam, valproate, lamotrigine, topiramate, lacosamide, zonisamide, perampanel, and eslicarbazepine—in focal or generalized epilepsies.^21^, ^22^, ^23^, ^24^, ^25^, ^26^, ^27^, ^28^ Unfortunately, information on the use of these ASMs, and especially CNB in DEEs, remains limited, with only three case series of four LGS (two of them 50% responders),^29^ another four LGS (two of them 50% responders),^30^ and six DEE (five of them 50% responders) patients.^31^ Overall, our results are comparable to retention rates observed in studies on other ASMs used in DEE (predominantly in LGS and DS), such as brivaracetam (65%),^32^ felbamate (50%),^33^, ^34^ levetiracetam (47%),^35^ perampanel (46%),^36^ topiramate (40%–47.8%),^37^, ^38^ and zonisamide (53.3%).^39^ Recently approved ASMs for the treatment of LGS are rufinamide, cannabidiol, and fenfluramine, with 50% responder rates of 31.1%–47.9% for rufinamide,^40^, ^41^, ^42^ 44.0%–50.0% for cannabidiol,^43^, ^44^ and 25.0% for fenfluramine.^45^ Interestingly, neurostimulation of the vagus nerve has demonstrated a comparable 50% responder rate of more than 40%.^46^, ^47^, ^48^ Overall, responder rates ≥ 50% in DEEs appear to be achievable in 30%–45% of treated patients, with specific ASMs showing greater effectiveness for certain seizure types, such as perampanel or fenfluramine for GTCS.^45^, ^49^

Regarding efficacy, no significant differences were observed between adults and children or adolescents. Furthermore, there was no difference found in other characteristics like target dose, number of previously failed ASMs, or number of concomitant ASMs. However, the limited number of patients makes the statistical analysis of response predictors difficult.

The mode of action of CNB as a sodium channel blocker raises concerns that it might exacerbate seizures in DS. Our single DS patient experienced a significant reduction in seizure frequency following CNB treatment, and this aligns with a previous report of four adult patients with DS who also benefited from CNB.^50^ Additionally, a 25‐year‐old female with DS was reported to achieve complete remission of GTCS.^30^ This might reflect previous observations that sodium channel blockers, including lamotrigine, can be beneficial later in the course of the disease, such as in adult patients with DS.^20^ In the author's opinion, sodium channel blockers should be avoided in children with DS, as evidence suggests they may trigger status epilepticus and lead to further cognitive decline.

The titration of CNB to a mean daily dose of 100 mg in children and 200 mg in adults suggests that underdosing is unlikely in this study. The C013 and C017 randomized trials demonstrated a clear dose–response relationship for CNB in adults,^4^, ^6^ with higher doses yielding greater efficacy, which is stated in the prescribing information that recommends a target dose of 200 mg. In our analysis, physicians aimed primarily for 100 or 200 mg as the typical target dose. This is in line with an observational study that showed that CNB is already effective at the low dose of 100 mg/day in refractory focal epilepsy.^51^ The 3‐month follow‐up point may not fully reflect the steady‐state efficacy of CNB, as the recommended target dose of 200 mg/day requires a titration period of at least 12 weeks to minimize the risk of severe cutaneous adverse reactions. Additionally, with a half‐life of approximately 55 h, cenobamate requires approximately 2 weeks (five half‐lives) after reaching the target dose to achieve steady‐state plasma levels. In our study, the median time to reach the target dose was 70 days, meaning that many patients may not have been at steady‐state plasma levels by the 3‐month assessment point.

CNB was generally well tolerated, with 24 (58.5%) patients reporting TEAEs, leading to the withdrawal of seven patients (17.1%). The rates of psychobehavioral TEAEs were low, at 12.2%, compared to the adverse events profiles in DEEs of other ASMs such as brivaracetam, perampanel, topiramate, or zonisamide.^52^ TEAEs like sedation, dizziness, ataxia, and blurred/double vision are common when CNB is used with other ASMs, especially sodium channel‐blocking agents.^53^ To reduce these risks, it is recommended to adjust the doses of sodium channel‐blocking agents when coadministered with CNB. In addition, CNB may inhibit the metabolism of clobazam, leading to increased sedation, which can be mitigated by reducing the dose of clobazam.

There were several strengths, as well as notable limitations, in this study. Strengths included the analysis of a comprehensive range of efficacy outcomes and a relatively large population size for a rare disease. Additionally, the inclusion of a wide range of prior and concomitant ASMs reflects the current treatment landscape for DEEs, such as LGS. However, the retrospective, uncontrolled, and open‐label design limits the reliability of the findings and introduces potential biases. For instance, clinicians were not blinded to the treatment, which could have influenced partially subjective outcomes like CGI‐C. Although clinicians were trained in its application, CGI‐C assessments were only conducted by clinicians, without additional perspectives from parents or caregivers. This may have overlooked valuable insights into the impact of treatment on daily life. The study's interview‐based design could have led to underreporting of TEAEs. Nonetheless, interviews conducted by epilepsy specialists and the prompt documentation of events likely mitigated this risk. Additionally, longer follow‐up is needed to assess sustained efficacy and safety. Further studies are also required to explore the treatment's impact on cognitive and behavioral impairments and overall quality of life.

## 5 CONCLUSIONS

We conclude that CNB can achieve a meaningful reduction in seizure frequency, including seizure freedom, in patients with DEEs in clinical practice. The pattern of TEAEs appears to be comparable to other frequently used ASMs.

## AUTHOR CONTRIBUTIONS

Elisa Buhleier, Susanne Schubert‐Bast, Felix Rosenow, and Adam Strzelczyk developed the idea for this study. Elisa Buhleier, Susanne Schubert‐Bast, Susanne Knake, Felix von Podewils, Hajo M. Hamer, Nico Melzer, Gerhard Kurlemann, Kerstin Alexandra Klotz, and Adam Strzelczyk participated in the recruitment of patients and data collection. Adam Strzelczyk supervised the study. Elisa Buhleier and Adam Strzelczyk conceived the paper and performed the statistical analysis. Elisa Buhleier, Laurent M. Willems, and Adam Strzelczyk created the charts and figures. Elisa Buhleier, Susanne Schubert‐Bast, Susanne Knake, Felix von Podewils, Hajo M. Hamer, Nico Melzer, Gerhard Kurlemann, Kerstin Alexandra Klotz, Laurent M. Willems, Felix Rosenow, Andreas Brunklaus, and Adam Strzelczyk wrote the paper, discussed the results, contributed to the final manuscript, and approved the final manuscript for publication.

## CONFLICT OF INTEREST STATEMENT

E.B. and L.M.W. do not report any conflicts of interest. S.S.‐B. has received personal fees and grants from Angelini Pharma, Biocodex, Desitin Arzneimittel, Eisai, Jazz Pharmaceuticals, Marinus, Takeda, and UCB Pharma. S.K. has received speaker's honoraria from Bial, Destin Arzneimittel, Eisai, Jazz Pharma, Merck Serono, and UCB. F.v.P. has received personal fees and grants from Angelini Pharma, Desitin Arzneimittel, Eisai, Jazz Pharmaceuticals, UCB Pharma, Nutricia Milupa, Neuraxpharm, and Bial. H.M.H. has served on the scientific advisory boards of Angelini, UniQure, Eisai, GW/Jazz, and UCB Pharma. He has served on the speakers' bureaus of or received unrestricted grants from Angelini, Ad‐Tech, Alnylam, Bracco, Desitin, Eisai, Jazz, LivaNova, Nihon Kohden, Pfizer, and UCB Pharma. N.M. has received honoraria for lecturing and travel expenses for attending meetings from Biogen Idec, GlaxoSmithKline, Teva, Novartis Pharma, Bayer Healthcare, Genzyme, Alexion Pharmaceuticals, Fresenius Medical Care, Diamed, UCB Pharma, Angelini Pharma, Bial, and Sanofi‐Aventis, has received royalties for consulting from UCB Pharma, Alexion Pharmaceuticals, and Sanofi‐Aventis, and has received financial research support from Euroimmun, Fresenius Medical Care, Diamed, Alexion Pharmaceuticals, and Novartis Pharma. G.K. has received speaker's honoraria from Angelini Pharma, Desitin Arzneimittel, Eisai, Jazz Pharmaceuticals, Takeda, UCB Pharma, Neuraxpharm, Stada Arzneimittel, Precisis, and Alexion Pharmaceuticals. K.A.K. has received speaker's honoraria from Biocodex, Desitin Arzneimittel, Eisai, Jazz Pharmaceuticals, and UCB Pharma. F.R. has received personal fees from Angelini Pharma, Desitin Arzneimittel, Eisai, Jazz Pharmaceuticals, Roche Pharma, Stoke Therapeutics, and UCB Pharma and grants from the Detlev‐Wrobel‐Fonds for Epilepsy Research, the Deutsche Forschungsgemeinschaft, the Federal Ministry of Education and Research, the LOEWE Program of the State of Hesse, and the European Union. A.B. has received honoraria for presenting at educational events, serving on advisory boards, and consultancy work for Biocodex, Encoded Therapeutics, Jazz/GW Pharma, Servier, Stoke Therapeutics, and UCB/Zogenix. A.S. has received personal fees and grants from Angelini Pharma, Biocodex, Desitin Arzneimittel, Eisai, Jazz Pharmaceuticals, Longboard, Neuraxpharm, Takeda, UCB Pharma, and UNEEG Medical. We confirm that we have read the Journal's position on issues involved in ethical publication and affirm that this report is consistent with those guidelines.

## Supporting information


Figure S1.

## Data Availability

The data for this study are available from the corresponding author upon reasonable request.
