# Supplementary figures and images for: A multicenter cohort study on the efficacy, retention, and tolerability of cenobamate in patients with developmental and epileptic encephalopathies
Source: Epilepsia. 2025 Feb 11;66(5):1519–28. doi: 10.1111/epi.18308 (PMC12097476; doi:10.1111/epi.18308)

Supplementary Figure 1A

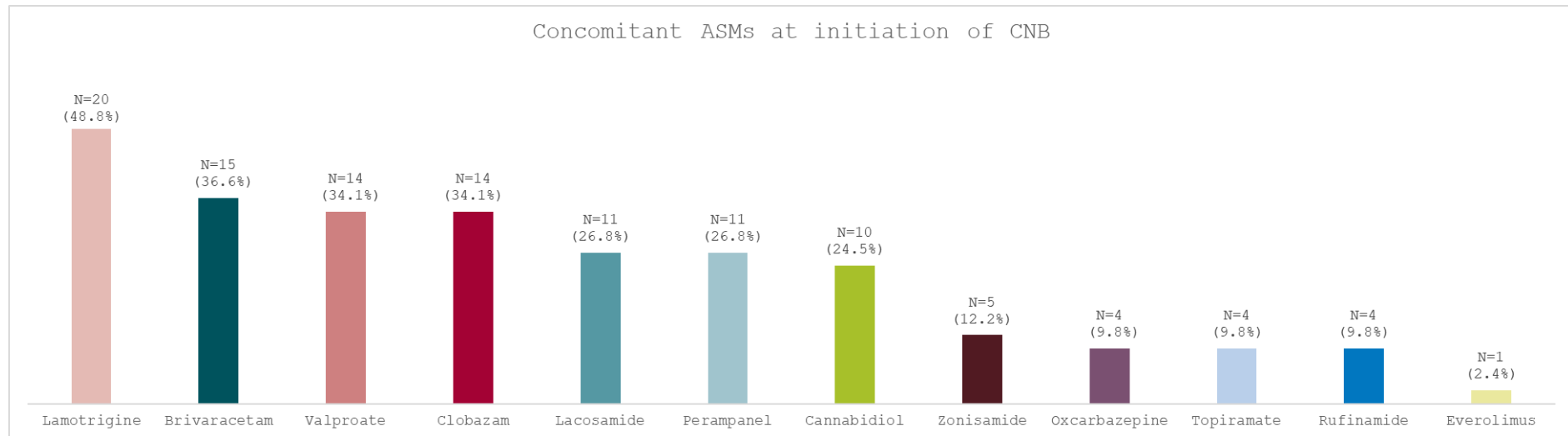

Supplementary Figure 1B

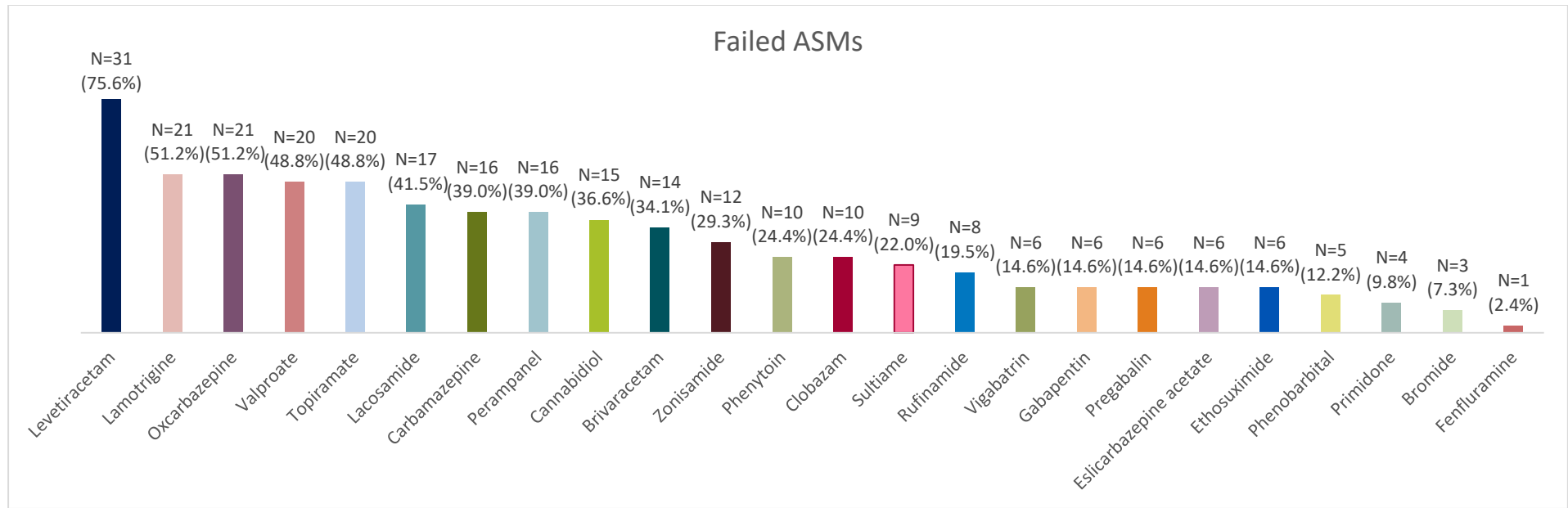

Supplement: Supplementary file 1 — Figure S1. [file EPI-66-1519-s001.pdf]
